# Supplementary material for: Clinical Outcomes and Oral Health-Related Quality of Life after Periodontal Treatment with Community Health Worker Strategy in Patients with Type 2 Diabetes: A Randomized Controlled Study
Source: Int J Environ Res Public Health. 2021 Aug 7;18(16):8371. doi: 10.3390/ijerph18168371 (PMC8394731; doi:10.3390/ijerph18168371)
Supplement: Supplementary file 1 [file ijerph-18-08371-s001.zip › ijerph-1269574-SI.pdf]

Table S1. Contents of periodontal care curriculum.

1. Toothbrushing tools and methods	
**1.1**	Choice of toothbrush
1.2	Appropriate toothbrushing methods (Bass / Modified Bass technique)
1.3	Other commonly used toothbrushing tools
2. Treatment and care of periodontal disease	
2.1	Process of periodontal treatment
2.2	Oral health self-care methods
2.3	Feedback of toothbrushing skill (with plaque disclosing agents)
3. Oral health knowledge related to diabetes mellitus	
3.1	The correlation between diabetes and periodontal disease
3.2	Common oral health problems in diabetes
3.3	Feedback of toothbrushing skill (with plaque disclosing agents)
3.4	Provide self-check method of teeth cleanliness by plaque disclosing agent
4. Overall review	
4.1	Review the content of the previous curriculum and emphasize the key points
4.2	Feedback of toothbrushing skill (with plaque disclosing agents)
